# Preparation of Oil Sludge-Based Activated Carbon Loaded with CuO/Fe_2_O_3_ and Its Mechanism in Activating Peroxymonosulfate for p-Nitrophenol Degradation

**DOI:** 10.3390/molecules31162755

**Published:** 2026-08-07

**Authors:** Hongqiang Yuan, Zheyuan Zhan, Ying Zhang, Yue Ran, Chaofan Tan, Jialian Xie

**Affiliations:** School of Urban Construction, Yangtze University, Jingzhou 434023, China; hqyuan@yangtzeu.edu.cn (H.Y.);

**Keywords:** oil sludge, activated carbon, copper oxide doping, peroxymonosulfate (PMS), real water matrices

## Abstract

In this study, oil sludge was utilized as a carbon source to prepare a carbon-based catalyst. By leveraging the oil sludge’s inherent iron content and subsequently introducing exogenous copper, a CuO/Fe_2_O_3_/carbon composite material (OSC-0.75Cu) was synthesized for the activation of peroxymonosulfate (PMS) to degrade p-nitrophenol. The OSC-0.75Cu/PMS system achieved a degradation efficiency of 92.19%, which was 2.91 times that of the OSC/PMS system (31.71%), and its reaction rate constant (k_obs_) was 8.9 times greater. Furthermore, in simulated water matrices (containing anions and fulvic acid) and three real water matrices (rainwater, moat water, and Yangtze River water), the OSC-0.75Cu/PMS systemachieved PNP degradation efficiencies ranging from 71.63% to 76.36%. The degradation of p-nitrophenol in this system involves three primary reactive species: hydroxyl radicals (•OH), sulfate radicals (•SO_4_^−^), and singlet oxygen (^1^O_2_), among which ^1^O_2_ plays the dominant role. The coexistence of Cu and Fe species may facilitate surface redox reactions during PMS activation, while defect-related oxygen sites may contribute to PMS adsorption and activation and the generation of ^1^O_2_. This study provides a novel strategy that enables the resource utilization of oil sludge while simultaneously addressing environmental remediation challenges.

## 1. Introduction

Oil sludge is one of the most common wastes in the petroleum industry and also represents an important biomass resource [1]. Oil sludge harbors a substantial load of hazardous and toxic pollutants, posing significant environmental risks if not managed through proper treatment [2]. Treatment methods for oil sludge include incineration, advanced oxidation, biological treatment, microwave irradiation, and pyrolysis [3]. Among these, pyrolysis technology is one of the most valuable approaches for oil sludge utilization [4], as it achieves volume reduction while producing a carbon-rich residue known as oil sludge carbon (OSC). With its low cost, high carbon content, and wide availability, OSC is a promising carbon material for effective adsorptive removal of harmful pollutants from water [5]. However, relying solely on adsorption cannot degrade pollutants, which may lead to secondary pollution.

As an emerging oxidation technology, the peroxymonosulfate activation system has recently garnered increasing attention in both research and practical water treatment applications. Compared with •OH, •SO_4_^−^ exhibits a considerably higher standard redox potential and a longer half-life [6,7]. Therefore, it can be used to decompose and mineralize the majority of refractory organic pollutants [8]. Various methods for PMS activation exist, such as thermal activation [9], ultraviolet (UV) light [10], and carbon-based material activation. Among these, transition metal-based materials (e.g., Cu, Fe, and Co) have become research hotspots due to their high efficiency [11]. Compared with cobalt, iron and copper have attracted greater attention from both environmental and economic perspectives [12,13]. In particular, copper–iron-loaded carbon-based materials exhibit higher PMS activation efficiency than corresponding single-metal systems [14]. This enhancement is attributed to the difference in standard redox potentials between Cu and Fe. Specifically, Cu facilitates valence cycling and electron transfer, thus enhancing the catalyst’s activating ability [15]. However, metal catalysts often suffer from significant metal leaching, which may cause secondary pollution during water treatment. To address this issue, carbon materials can be used as supports to stabilize metal species and suppress leaching. Previous studies have explored supports such as carbon nanotubes [16], graphene [17], and biochar [18]. Utilizing OSC as a support not only replaces high-cost carbon materials but also enables a sustainable strategy for waste reutilization.

p-Nitrophenol (PNP) was selected as the target pollutant because it is a priority nitroaromatic contaminant with considerable biological toxicity. PNP may undergo biotransformation in organisms, leading to hormonal disruption and cell death [19,20]. Furthermore, its strong antioxidant capacity makes it difficult to remove through conventional chemical oxidation and biological processes [20]. Therefore, PNP serves as a suitable model pollutant for evaluating the catalytic performance and reaction mechanism of the OSC-0.75Cu/PMS system.

In light of the above considerations, this study aimed to develop an oil sludge-derived CuO/Fe_2_O_3_/carbon catalyst for PMS activation. To the best of our knowledge, this is the first attempt to utilize the inherent iron species in oil sludge, together with externally introduced copper, to construct such a catalyst for PMS activation. The effects of copper loading, PMS dosage, catalyst dosage, initial PNP concentration, temperature, and pH on catalytic performance were systematically investigated. The influences of common inorganic anions, fulvic acid, and three real water matrices were further evaluated to assess the robustness of the system against complex water constituents. Reactive species, surface redox changes, transformation products, and metal leaching were analyzed to elucidate the underlying mechanisms of PMS activation and PNP degradation.

## 2. Results

### 2.1. Morphology and Characterization

**Figure 1:**
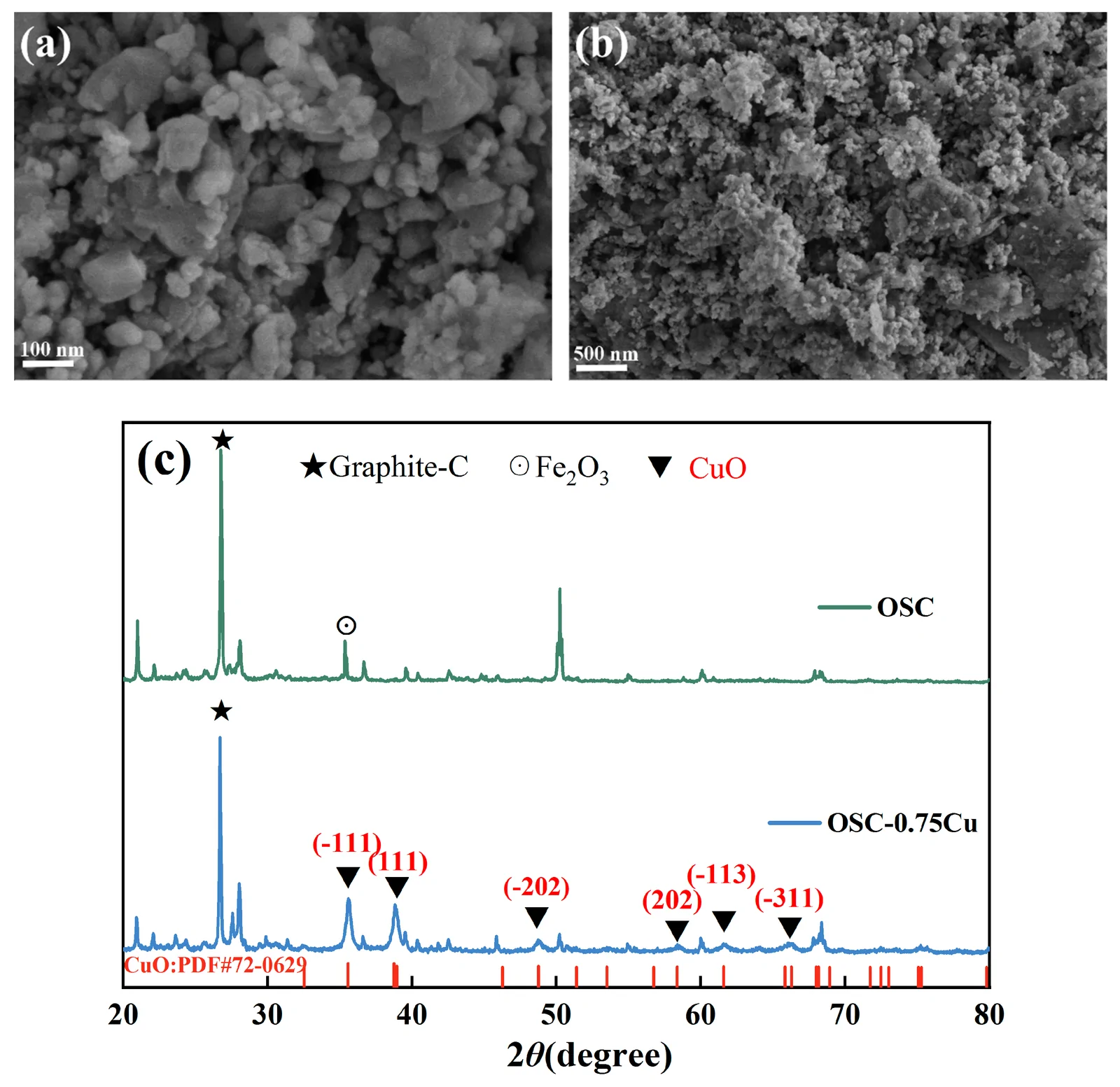
Scanning electron microscope (SEM) images of OSC-0.75Cu: (**a**) 100 k× and (**b**) 20 k×. (**c**) XRD patterns of OSC and OSC-0.75Cu.

The XRD patterns of OSC and OSC-0.75Cu are presented in Figure 1c. In the OSC pattern, a diffraction peak corresponding to Fe_2_O_3_ was observed at 2θ = 35.36° [21], indicating that the oil sludge inherently contains iron, which formed Fe_2_O_3_ during high-temperature pyrolysis [22]. Both OSC and OSC-0.75Cu exhibited a strong diffraction peak at 2θ = 26°, corresponding to the (002) crystal plane of graphite, suggesting that both samples possess a graphite structure. In the OSC-0.75Cu pattern, new characteristic peaks appeared at 2θ = 35.26°, 38.75°, 48.75°, 58.35°, 61.57°, and 66.28°, which match well with the characteristic peaks of copper oxide (PDF#72-0629), corresponding to the (−111), (111), (−202), (202), (−113), and (−311) crystal planes, respectively. The diffraction peaks of copper oxide exhibited high crystallinity, indicating that copper oxide was successfully loaded onto the OSC surface, forming a CuO/Fe_2_O_3_/OSC composite catalyst.

The morphology of OSC-0.75Cu was analyzed by SEM imaging (Figure 1a,b). Numerous fine particles were observed on the surface, which are attributed to copper oxide and iron oxide particles. Additionally, abundant porous structures were visible on the catalyst surface, indicating that the re-calcination process at 500 °C did not compromise the favorable pore structure.

**Figure 2:**
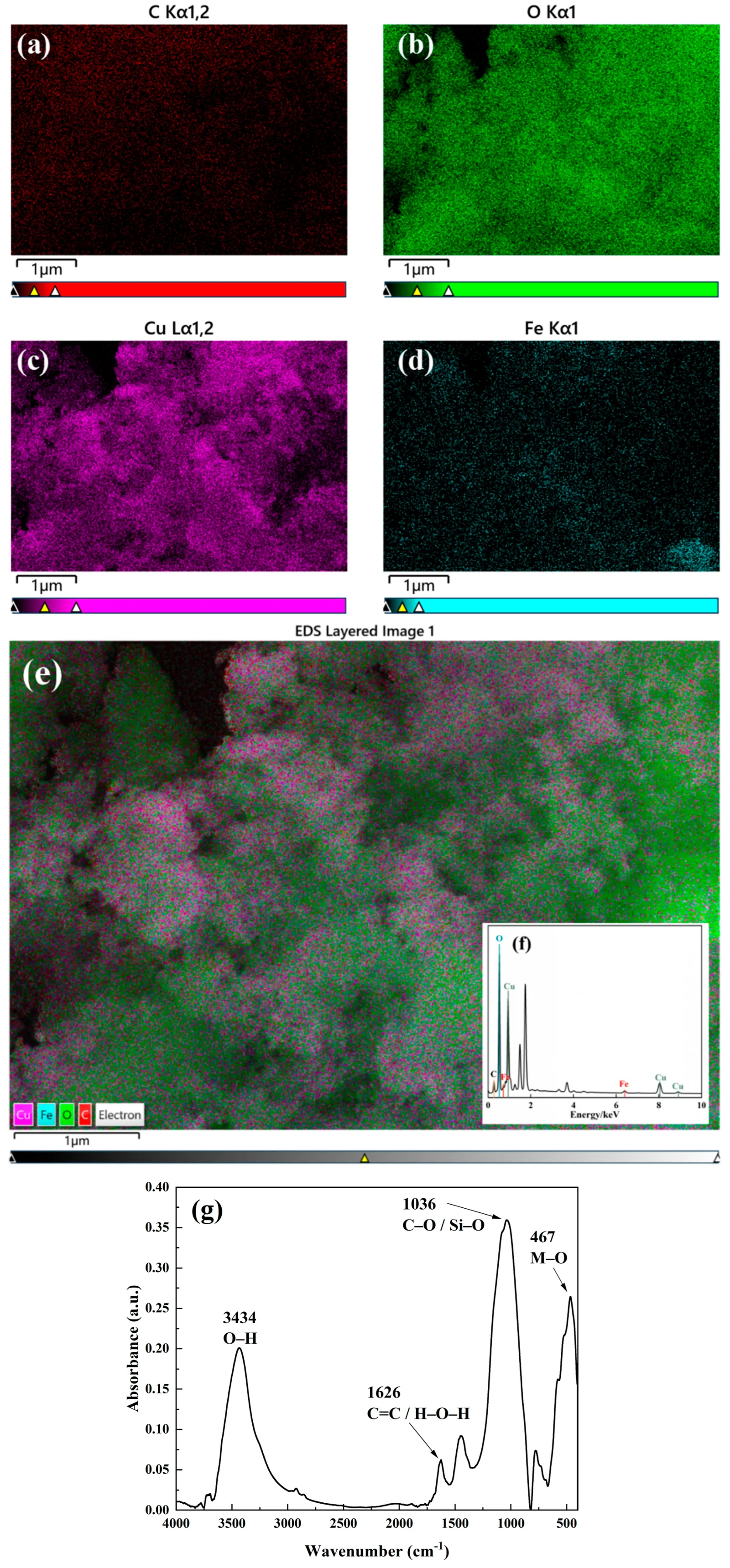
EDS elemental mapping images of OSC-0.75Cu: (**a**) C, (**b**) O, (**c**) Cu, (**d**) Fe, and (**e**) elemental overlay map. (**f**) EDS spectrum and (**g**) FTIR spectrum of fresh OSC-0.75Cu.

The EDS spectra and elemental mapping results (Figure 2a–f) confirmed the presence and spatial distribution of O, C, Fe, and Cu in OSC-0.75Cu. As shown in Figure 2c, the copper element was uniformly distributed, suggesting that the preparation method enabled the uniform dispersion of copper oxide on the carbon support. Since no external iron source was added, the formation of iron oxide depended on the intrinsic iron present in the oil sludge; therefore, the iron distribution was relatively dispersed (Figure 2d). As shown in Figure 2g, the FTIR spectrum of OSC-0.75Cu exhibited a broad absorption band at approximately 3434 cm^−1^, which was assigned to the O–H stretching vibration of surface hydroxyl groups and adsorbed water. The band at approximately 1626 cm^−1^ was associated with aromatic C=C vibrations and may partially overlap with the H–O–H bending vibration of adsorbed water. The prominent band at approximately 1036 cm^−1^ was mainly attributed to C–O-containing surface groups, although a contribution from Si–O species in the mineral fraction could not be excluded. In addition, the band at approximately 467 cm^−1^ was associated with metal–oxygen vibrations. The oxygen-containing functional groups on the catalyst surface will be further analyzed in the subsequent XPS section.

### 2.2. Effects of Various Reaction Factors on PNP Degradation

#### 2.2.1. Effect of Copper Oxide Loading Ratios

**Figure 3:**
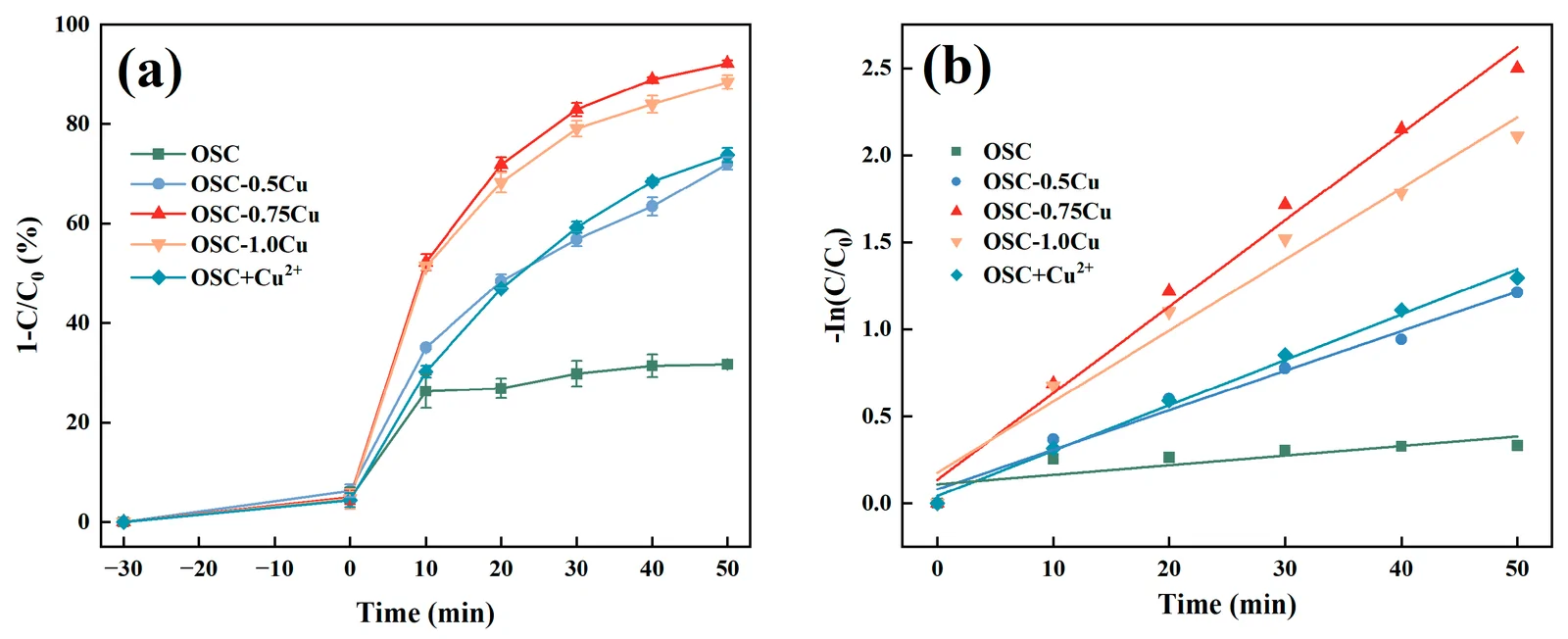
(**a**) PNP degradation curves of different catalysts; (**b**) corresponding pseudo-first-order kinetics fitting.

The catalytic performance of OSC-*X*Cu with different copper oxide loading ratios was evaluated for PNP degradation at a PMS concentration of 1.4 mmol/L (Figure 3). All OSC-*X*Cu catalysts exhibited significantly enhanced PMS activation capability. Pristine OSC achieved a PNP degradation efficiency of only 31.71%. Upon introducing the copper oxide, the efficiency increased markedly; OSC-0.75Cu reached a maximum degradation efficiency of 92.19% (a 2.91-fold increase over OSC), with a 9.03-fold higher k_obs_. The bimetallic system outperforms the single-metal system due to synergistic effects between copper oxide and iron oxide species anchored on the carbon matrix [18]. However, at higher copper oxide loading (OSC-1.0Cu), efficiency dropped slightly, likely due to the excessive coverage of active sites by copper oxide.

Control experiments involving the addition of Cu^2+^ ions (with an OSC-to-copper nitrate mass ratio of 1:0.75) to the OSC/PMS system (Figure 3) showed 75% degradation, which is lower than the 92.19% achieved by the heterogeneous OSC-0.75Cu/PMS system. This disparity highlights the synergistic interaction between CuO and the Fe_2_O_3_/carbon matrix, rather than simple homogeneous catalysis by leached ions.

#### 2.2.2. Effect of PMS Dosage

**Figure 4:**
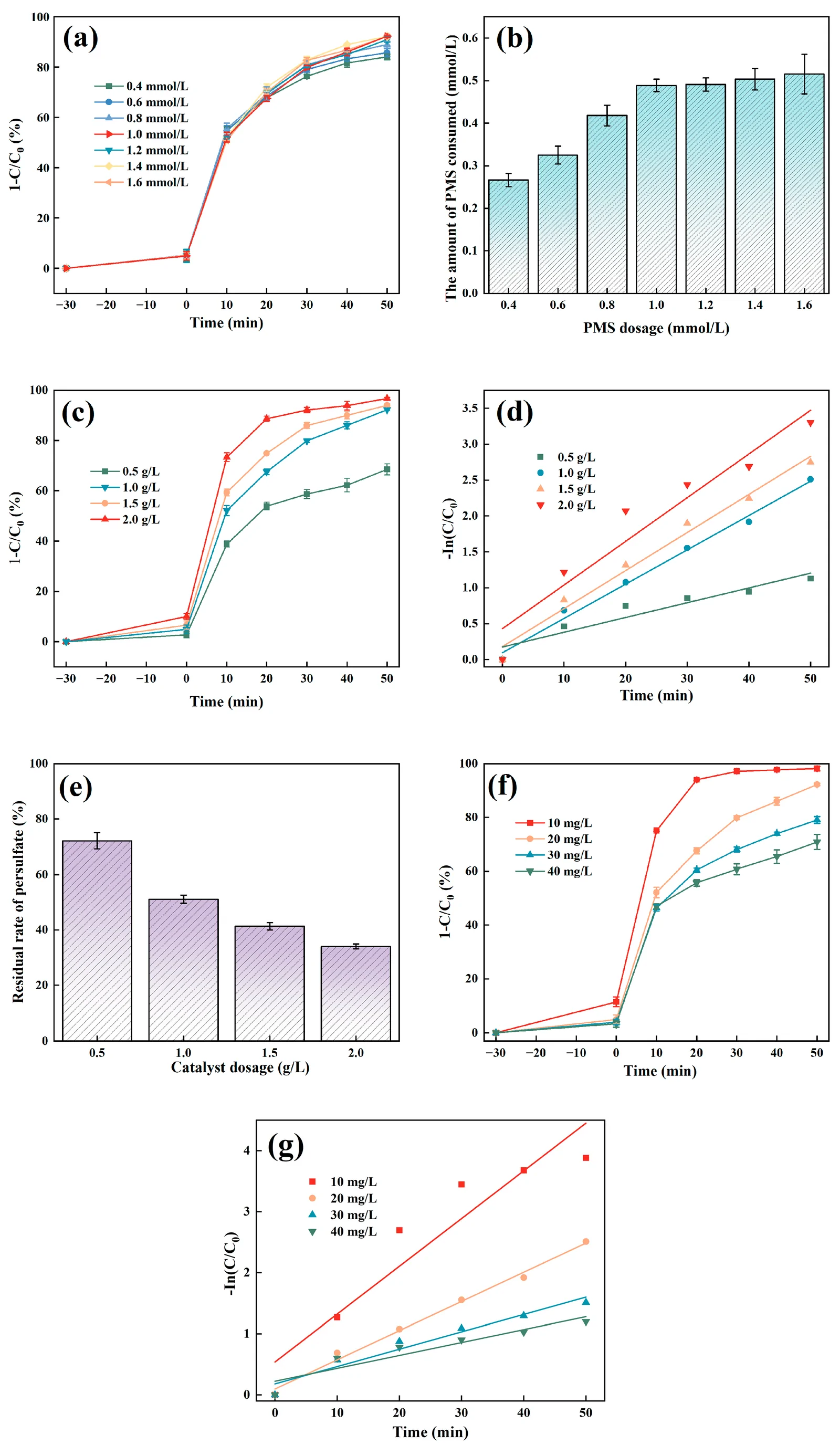
(**a**) Effect of PMS dosage on the degradation efficiency of PNP; (**b**) actual PMS consumption; (**c**) effect of catalyst dosage on the degradation efficiency of PNP; (**d**) corresponding pseudo-first-order kinetics fitting; (**e**) PMS residual rate as a function of catalyst dosage; (**f**) effect of initial PNP concentrations on the degradation efficiency of PNP; (**g**) corresponding pseudo-first-order kinetics fitting.

PNP degradation in the OSC-0.75Cu/PMS system was investigated across a PMS dosage range of 0.4–1.6 mmol/L. As shown in Figure 4a, PNP degradation efficiency rose from 84.05% to 92.26% as PMS dosage increased from 0.4 to 1.0 mmol/L. However, further increases in the PMS dosage resulted in negligible improvement. This behavior results from PMS serving as the precursor of reactive species; hence, increasing its concentration initially enhances reactive oxygen species generation. However, once the available catalytic active sites become saturated, further increases in PMS no longer promote additional reactive species formation. Indeed, further increasing PMS to 1.6 mmol/L resulted in minimal changes in the PNP degradation efficiency, whereas PMS consumption increased from 0.489 to 0.515 mmol/L (Figure 4b). This is likely because excess PMS undergoes side reactions, consuming reactive species and thereby reducing the overall oxidative degradation capacity of PMS [23,24]. Hence, 1 mmol/L PMS was chosen for the following experiments.

#### 2.2.3. Effect of Catalyst Dosage

The influence of OSC-0.75Cu dosage on PNP degradation is shown in Figure 4c. Increasing the catalyst dosage significantly enhanced degradation efficiency and reaction kinetics. After 50 min, catalyst dosages of 1.0–2.0 g/L achieved degradation efficiencies above 90% but only reached 68.52% at 0.5 g/L. The k_obs_ with 2.0 g/L (0.0608 min^−1^) was approximately three times that with 0.5 g/L (0.0206 min^−1^). These results are due to the increased OSC-0.75Cu catalyst dosage, which offers additional active sites for PMS activation to produce more reactive species and thereby improve the PNP degradation efficiency.

However, when the OSC-0.75Cu dosage exceeded 1.0 g/L, degradation efficiency improved slightly. The PNP degradation efficiency reached 92.19% at 1.0 g/L and 96.68% at 2.0 g/L. During the first ten minutes of the reaction, the system at 2.0 g/L exhibited a much faster PNP degradation rate than that at 1.0 g/L, but the difference diminished in the later stage (40–50 min). The residual PMS concentration after 50 min of reaction was determined using the iodometric method [25], as shown in Figure 4e. Higher catalyst dosages led to faster PMS decomposition and lower residual PMS concentrations. However, excessive PMS decomposition may produce reactive species in excess of what is required for effective pollutant degradation, leading to unproductive consumption of these species before they interact with PNP [26]. Therefore, considering both efficiency and cost, 1 g/L was adopted for subsequent studies under other conditions.

#### 2.2.4. Effect of Different PNP Concentrations

Figure 4f illustrates the influence of initial PNP concentration (10–40 mg/L) on OSC-0.75Cu/PMS degradation performance. Degradation efficiency declined from 98.17% to 70.86% as PNP concentration rose from 10 to 40 mg/L, with the k_obs_ dropping accordingly. This trend is due to increased competition for reactive species at higher pollutant concentrations, thereby reducing the degradation efficiency. Nevertheless, the absolute amount of PNP removed increased from 0.49 mg to 1.41 mg. This is because a higher PNP concentration increases the probability of collision between PNP molecules and reactive species, thus increasing the actual amount removed. These results demonstrate that the OSC-0.75Cu/PMS system is effective across a wide range of PNP concentrations.

#### 2.2.5. Effect of Temperature

**Figure 5:**
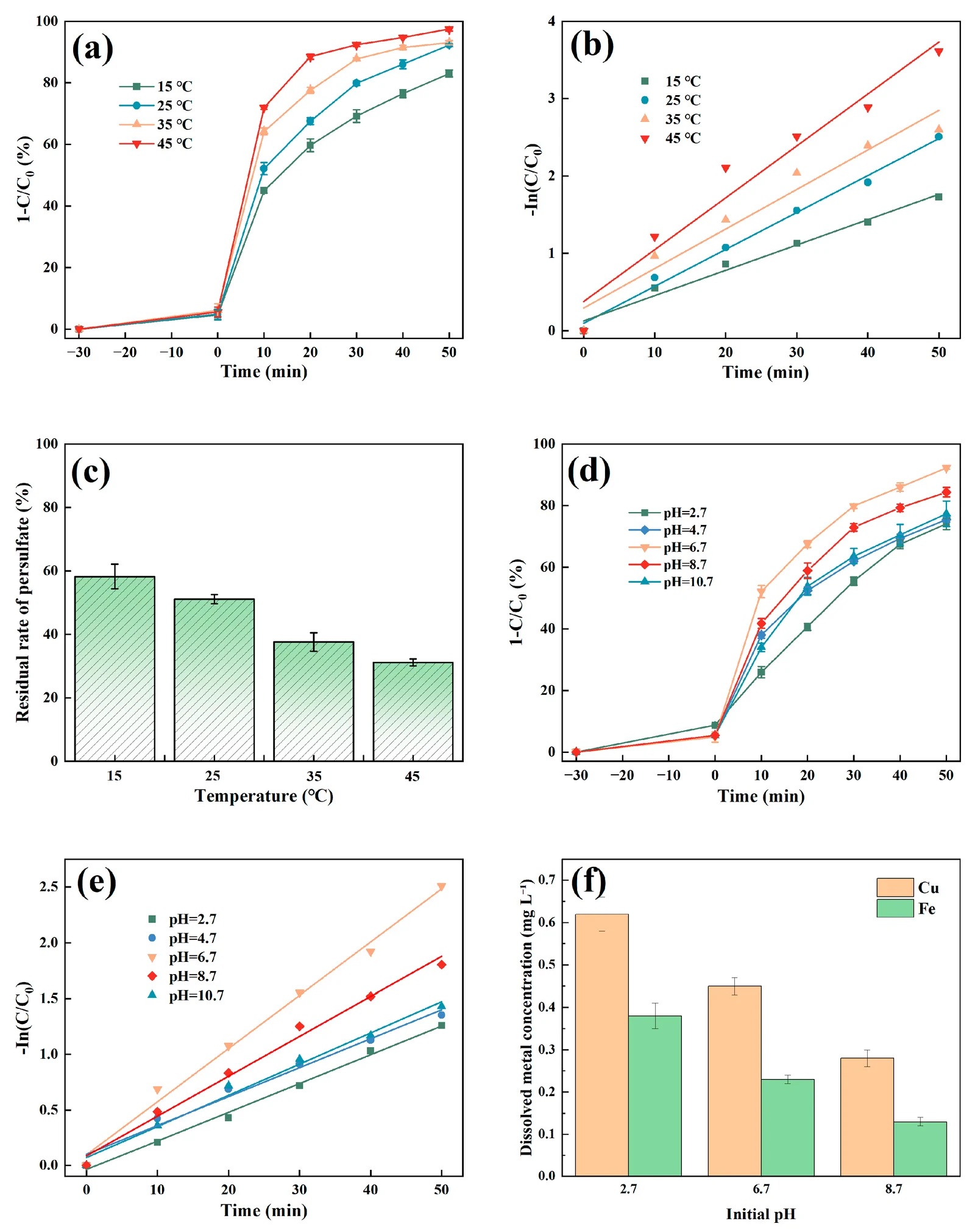
(**a**) Effect of temperature on the degradation efficiency of PNP; (**b**) corresponding pseudo-first-order kinetics fitting; (**c**) PMS residual rate at different temperatures; (**d**) effect of pH on the degradation efficiency of PNP; (**e**) corresponding pseudo-first-order kinetics fitting; (**f**) dissolved Cu and Fe concentrations under representative initial pH conditions after 50 min of reaction.

Studies have shown that temperature is an important factor in activating peroxymonosulfate, with thermal activation generally becoming more effective at higher temperatures. The effect of temperature on PNP degradation in the OSC-0.75Cu/PMS system is presented in Figure 5a. The reaction temperatures were set at 15, 25, 35, and 45 ± 2 °C. With increasing reaction temperature (15 to 45 °C), PNP degradation efficiency improved (83.05% to 97.45%), as did the k_obs_ (0.0327 to 0.0671 min^−1^), whereas residual PMS concentration declined (58.23% to 31.17%). This enhancement is attributed to the increased thermal energy, which accelerates PMS activation and raises the probability of collisions between the generated reactive species and the target pollutant, resulting in higher PNP degradation efficiency.

#### 2.2.6. Effect of pH

The effect of pH on PNP degradation in the OSC-0.75Cu/PMS system was investigated. According to Figure 5d, raising the pH from 2.7 to 6.7 enhanced both PNP degradation efficiency (from 74.07% to 92.56%) and k_obs_ (from 0.0259 to 0.0477 min^−1^). The lower degradation efficiency under acidic conditions may be partly associated with enhanced dissolution of surface metal species, which can influence PMS activation [27,28], as further supported by the Cu and Fe leaching results shown in Figure 5f. As the pH rose from 6.7 to 10.7, PNP degradation efficiency declined from 92.26% to 77.37%; correspondingly, the k_obs_ dropped from 0.0477 to 0.0281 min^−1^. This may be because under alkaline conditions, PMS readily decomposes into SO_5_^2−^, which hinders the generation of reactive species [29,30]. To examine whether metal leaching contributed to the pH-dependent catalytic performance, the dissolved Cu and Fe concentrations were measured under representative acidic, near-neutral, and alkaline conditions. As shown in Figure 5f, the dissolved Cu and Fe concentrations at pH 2.7 were 0.62 ± 0.04 and 0.38 ± 0.03 mg L^−1^, respectively, which were higher than those measured at pH 6.7 (0.45 ± 0.02 and 0.23 ± 0.01 mg L^−1^) and pH 8.7 (0.28 ± 0.02 and 0.13 ± 0.01 mg L^−1^). These results indicate that acidic conditions enhanced the dissolution of surface metal species. Therefore, increased metal leaching may partly contribute to the reduced catalytic performance under acidic conditions, although changes in PMS speciation and the catalyst surface environment may also be involved.

### 2.3. Effects of Simulated Water Matrices

#### 2.3.1. Effect of Inorganic Anions

**Figure 6:**
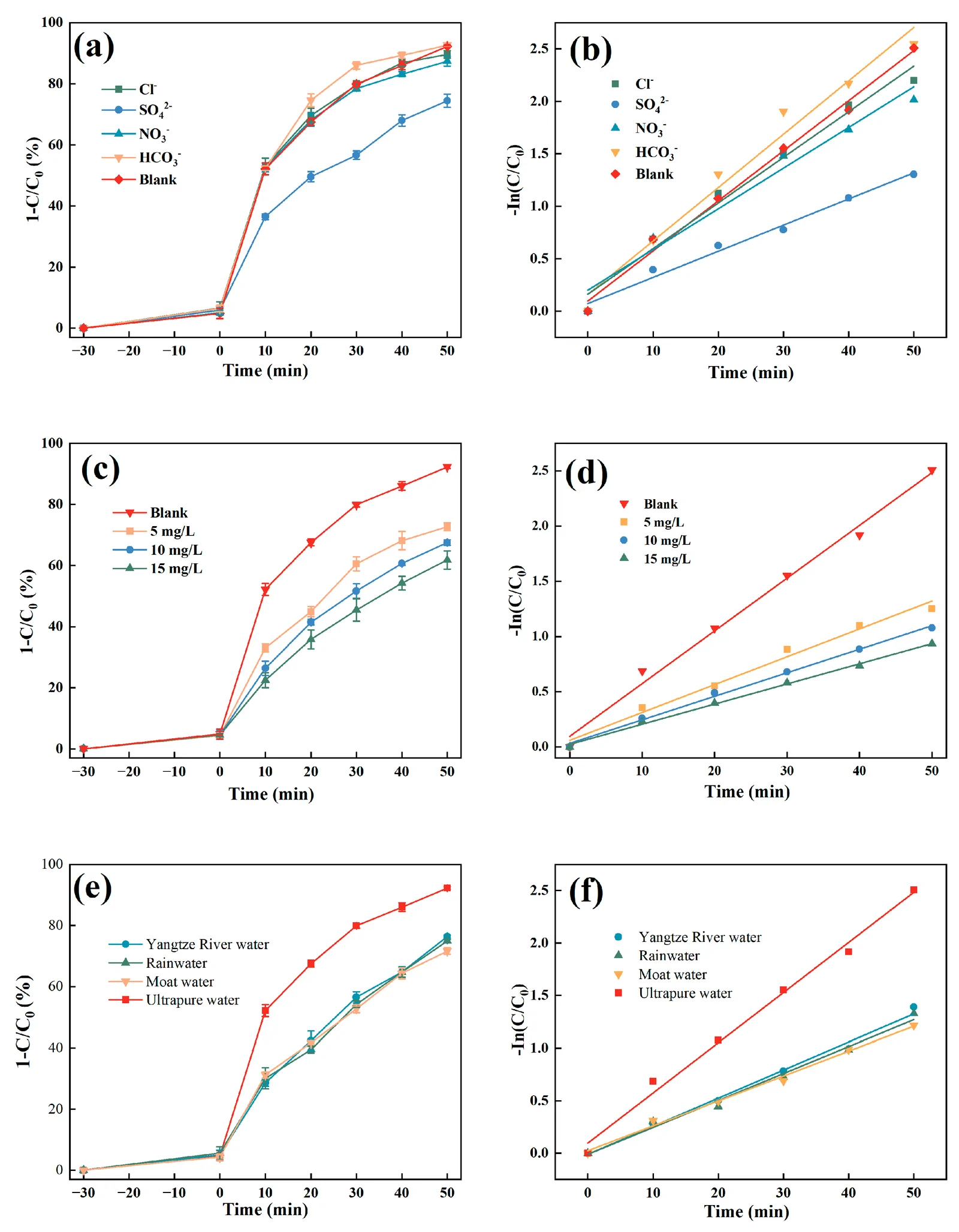
(**a**) Effect of different anions on the degradation of PNP; (**b**) corresponding pseudo-first-order kinetics fitting; (**c**) effect of fulvic acid on the degradation of PNP; (**d**) corresponding pseudo-first-order kinetics fitting; (**e**) effect of different real water matrices on the degradation of PNP; (**f**) corresponding pseudo-first-order kinetics fitting.

In real wastewater, various organic compounds and inorganic ions can affect the degradation efficiency of organic pollutants. The effects of common anions (Cl^−^, SO_4_^2−^, NO_3_^−^, and HCO_3_^−^, 10 mmol/L) on PNP degradation in the OSC-0.75Cu/PMS system are shown in Figure 6a. Cl^−^ slightly inhibited PNP degradation by reacting with •SO_4_^−^ to form secondary chlorine radicals with lower redox potentials [31] (Equations (1)–(4)). NO_3_^−^ also inhibited degradation, because it reacts with •SO_4_^−^ to form radicals with lower oxidation capacity (Equation (5)) [32]. The inhibitory effect of SO_4_^2−^ on PNP degradation is mainly attributed to its participation in reversible radical reactions [33].

Studies have also shown that HCO_3_^−^ can accelerate pollutant degradation in both homogeneous and heterogeneous AOPs [34,35], which is attributed to the asymmetric structure of PMS, making it prone to activation by HCO_3_^−^ and leading to rapid decomposition [36]. However, upon the addition of 10 mmol/L HCO_3_^−^, PNP degradation efficiency improved only slightly (92.26% to 92.65%), indicating that the presence of HCO_3^−^_ had no statistically significant effect on the overall degradation in our study.

•SO_4_^−^ + Cl^−^ → •Cl + SO_4_^2−^, (1)

•Cl + Cl^−^ → •Cl_2_^−^, (2)

•ClOH^−^ + H^+^ → •Cl + H_2_O, (3)

•Cl_2_^−^ + H_2_O → •ClOH^−^ + Cl^−^ + H^+^, (4)

•SO_4_^−^ + NO_3_^−^ → •NO_3_ + SO_4_^2−^. (5)

#### 2.3.2. Effect of Fulvic Acid

Fulvic acid (FA) is commonly found in natural water bodies and has abundant functional groups, which may affect the degradation efficiency of PNP. Consequently, an FA-containing aqueous solution was employed to simulate natural aquatic environments and investigate its effect on PNP degradation by the OSC-0.75Cu/PMS system. As shown in Figure 6c, increasing the FA concentration from 0 to 15 mg/L reduced PNP degradation efficiency from 92.26% to 61.78% and k_obs_ from 0.0477 to 0.0182 min^−1^, indicating that FA exerted an inhibitory effect on PNP degradation. This inhibitory effect may be because of the abundant functional groups of FA, which act as scavengers for •SO_4_^−^ and •OH [37]. Additionally, the reactive species generated by the OSC-0.75Cu/PMS system may attack both PNP and FA simultaneously, resulting in competitive consumption and consequently reducing the degradation efficiency of PNP [38].

#### 2.3.3. Effect of Real Water Matrices

To evaluate the applicability of the OSC-0.75Cu/PMS system for PNP degradation in real water matrices, water samples were collected from the Yangtze River, the Jingzhou ancient city moat, and rainwater for this study. After collection, the samples were filtered to remove suspended solids. As shown in Figure 6e, the OSC-0.75Cu/PMS system retained appreciable degradation efficiency for PNP in real water matrices. Specifically, PNP degradation efficiency reached 76.36% in Yangtze River water, 74.97% in rainwater, and 71.63% in moat water. The lower PNP degradation efficiencies in real water matrices may be attributed to the combined effects of organic matter, inorganic ions, and other matrix constituents. Because detailed water-quality parameters were not measured, the individual contribution of each component could not be determined.

### 2.4. Reusability Experiment

**Figure 7:**
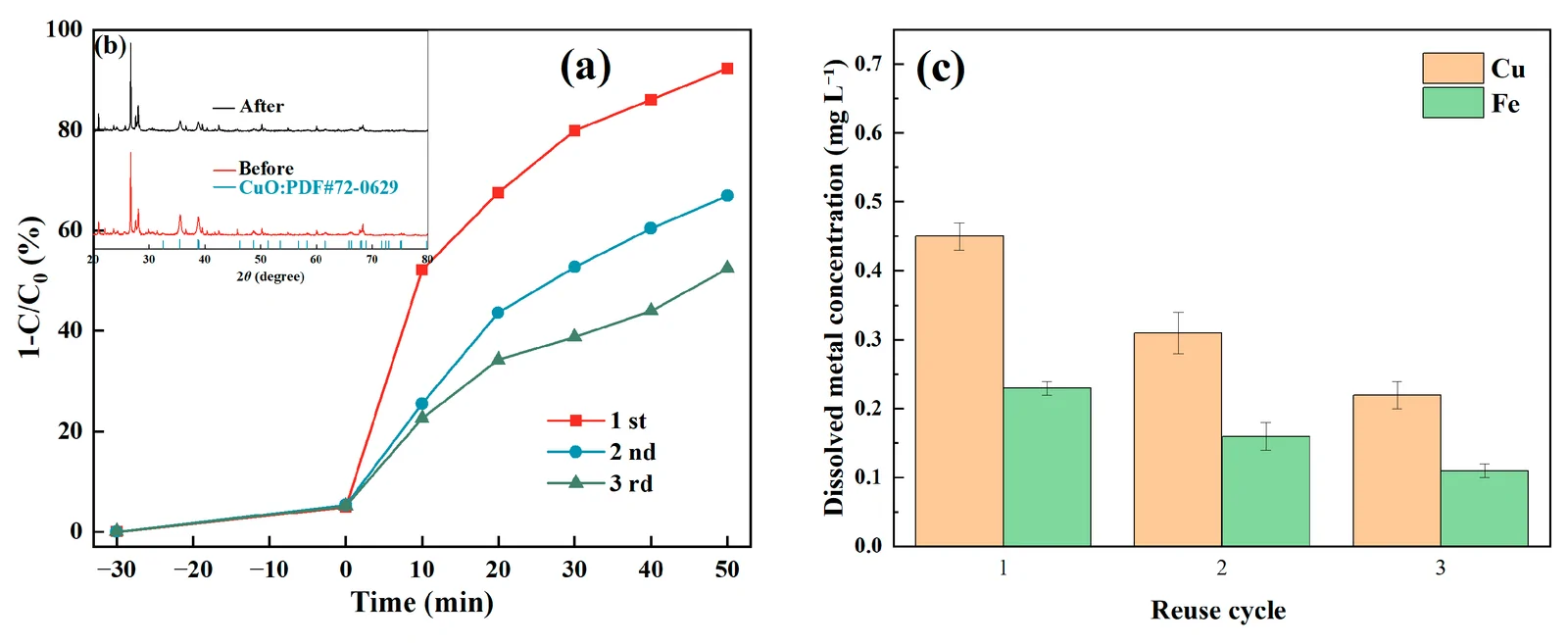
(**a**) Cycling experiment for PNP degradation using OSC-0.75Cu; (**b**) XRD patterns of OSC-0.75Cu before and after three cycles of degradation; (**c**) dissolved Cu and Fe concentrations after each reuse cycle.

The reusability of OSC-0.75Cu was evaluated through cycling experiments. The PNP degradation efficiency reached 92.26% in the first cycle, declined to 66.91% in the second cycle, and further dropped to 52.42% in the third cycle, demonstrating continuous deactivation (Figure 7a). The XRD patterns of the catalyst after three cycles are shown in Figure 7b. No significant peak shifts were observed in the XRD patterns after three cycles, suggesting that the main crystalline structure of OSC-0.75Cu was largely retained. However, the characteristic peaks of copper oxide significantly diminished, indicating partial copper loss. To quantitatively evaluate metal loss during catalyst reuse, dissolved Cu and Fe concentrations were measured after each reaction cycle. As shown in Figure 7c, the dissolved Cu concentration decreased from 0.45 ± 0.02 mg L^−1^ in the first cycle to 0.31 ± 0.03 and 0.22 ± 0.02 mg L^−1^ in the second and third cycles, respectively. The corresponding dissolved Fe concentrations decreased from 0.23 ± 0.01 to 0.16 ± 0.02 and 0.11 ± 0.01 mg L^−1^. This decreasing trend in metal release suggests that weakly bound surface metal species were preferentially leached during the initial cycles. Nevertheless, measurable Cu and Fe leaching persisted during reuse, indicating that metal loss may contribute to catalyst deactivation.

Therefore, the observed performance decline may be attributed to the following factors: (1) Leaching of metal ions weakened the metal cycling capability. Metal ions that leached during the reaction may have participated in homogeneous PMS activation in the solution. In addition, (2) deactivation may also result from blockage of active sites, caused by the adsorption of PNP and its by-products on the activated carbon surface, which hinders PMS access to the active sites.

Further work for enhancing the long-term stability of OSC-0.75Cu should consider improved metal immobilization and appropriate catalyst regeneration strategies.

## 3. Discussion

### 3.1. Analysis of Surface Elemental Valence States of the Catalyst

**Figure 8:**
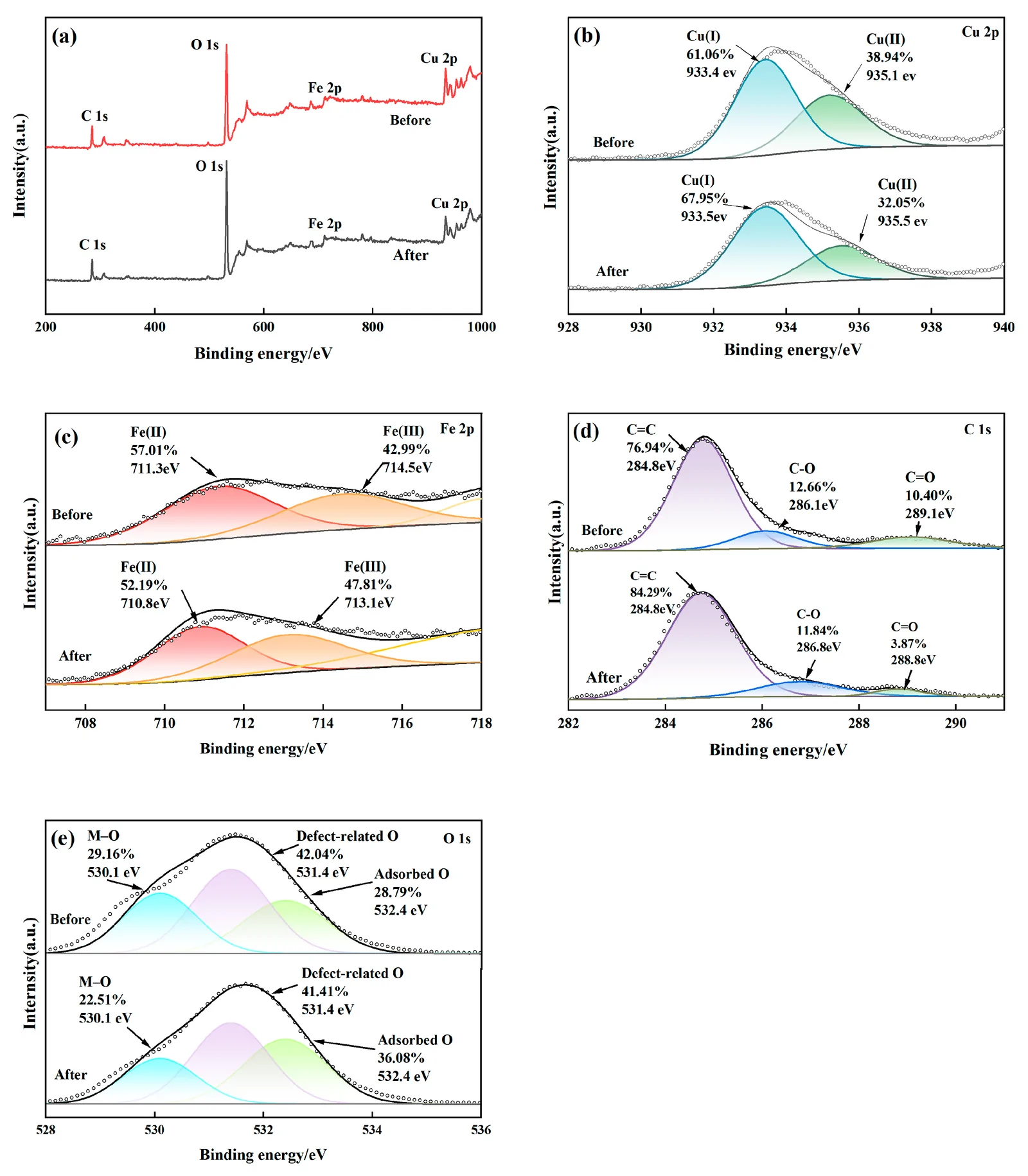
XPS spectra of OSC-0.75Cu before and after the reaction: (**a**) survey spectra, (**b**) Cu 2p, (**c**) Fe 2p, (**d**) C 1s, and (**e**) O 1s.

As shown in Figure 8a, OSC-0.75Cu contained O, C, Cu, and Fe elements, consistent with the SEM-EDS results. The same major elements remained detectable after the reaction, indicating that the principal elemental composition was retained. Figure 8b shows that the fresh OSC-0.75Cu exhibited Cu 2p peaks at 933.4 eV and 935.1 eV, corresponding to Cu(I) and Cu(II), respectively [39,40]. After 50 min of reaction, the Cu 2p spectrum showed peaks at 933.5 eV and 935.5 eV, corresponding to Cu(I) and Cu(II), respectively [41,42].

As shown in Figure 8c, the fresh OSC-0.75Cu exhibited Fe 2p peaks at 711.3 eV and 714.5 eV, corresponding to Fe(II) and Fe(III), respectively [43]. After 50 min of reaction, the Fe 2p spectrum showed peaks at 710.8 eV and 713.1 eV, both assigned to Fe(III) [44]. Based on peak fitting areas, the relative contents of Cu(I) increased from 61.06% to 67.95%, while Cu(II) showed a corresponding decrease from 38.94% to 32.05%. Similarly, the Fe(II) content declined from 57.01% to 52.19%, while Fe(III) rose from 42.99% to 47.81%. The changes in the Cu and Fe oxidation state distributions are consistent with a possible interfacial redox interaction between Cu and Fe species during PMS activation [45,46]. However, XPS provides indirect evidence, and the detailed electron-transfer pathway requires further investigation.

As shown in Figure 8d, fresh OSC-0.75Cu exhibited C 1s peaks at 284.8, 286.1, and 289.1 eV, corresponding to C=C, C—O, and C=O, respectively [47]. After 50 min of reaction, the C 1s binding energies were 284.8, 286.8, and 288.8 eV, with peaks corresponding to C=C, C—O, and C=O, respectively [48]. Based on peak fitting, C=O content dropped from 10.4% to 3.87%, whereas C—O slightly decreased from 12.6% to 11.8%, indicating greater involvement of C=O than C—O in the reaction. It is evident that C=O and C—O were involved in the degradation process. Both C=O and C—O can generate SO_4_^2−^, HSO_4_^−^, and ^1^O_2_ [49,50] (Equations (6) and (7)) in PMS activation, with ^1^O_2_ subsequently participating in the PNP degradation reaction.

As shown in Figure 8e, the O 1s spectra of OSC-0.75Cu were deconvoluted into three components centered at 530.1, 531.4, and 532.4 eV, which were assigned to metal–oxygen species (M–O), defect-related oxygen species (O_def_), and adsorbed oxygen species (O_ads_), respectively. For the fresh catalyst, the relative proportions of M–O, O_def_, and O_ads_ were 29.16%, 42.04%, and 28.79%, respectively. After the reaction, the M–O contribution decreased to 22.51%, whereas the proportions of O_def_ and O_ads_ changed to 41.41% and 36.08%, respectively. The appreciable proportion of O_ads_ in the fresh catalyst indicates the presence of abundant surface-accessible oxygen species. Previous studies have shown that surface-adsorbed oxygen species can facilitate PMS adsorption and activation and promote the generation of ^1^O_2_, thereby contributing to enhanced catalytic performance [51]. After the reaction, the increase in O_ads_ from 28.79% to 36.08% suggests a redistribution toward adsorbed oxygen species during PMS activation. Meanwhile, the persistently high proportion of O_def_ indicates that defect-related oxygen sites remain a prominent and relatively stable feature of the OSC-0.75Cu surface.

C=O + HSO_5_^−^ → C=O + SO_4_^2−^ + H^+^ + ^1^O_2_, (6)

C–O + HSO_5_^−^ → C–O + HSO_4_^−^ + ^1^O_2_. (7)

### 3.2. Quenching Experiments

To identify the reactive oxygen species (ROS) responsible for PNP removal by the OSC-0.75Cu/PMS system, quenching experiments were conducted. Specifically, BQ and HD were used to quench superoxide anion radicals (•O_2_^−^) and ^1^O_2_, respectively [52,53]; MeOH was used to quench both •OH and •SO_4_^−^, while TBA was used to quench •OH primarily [54,55].

**Figure 9:**
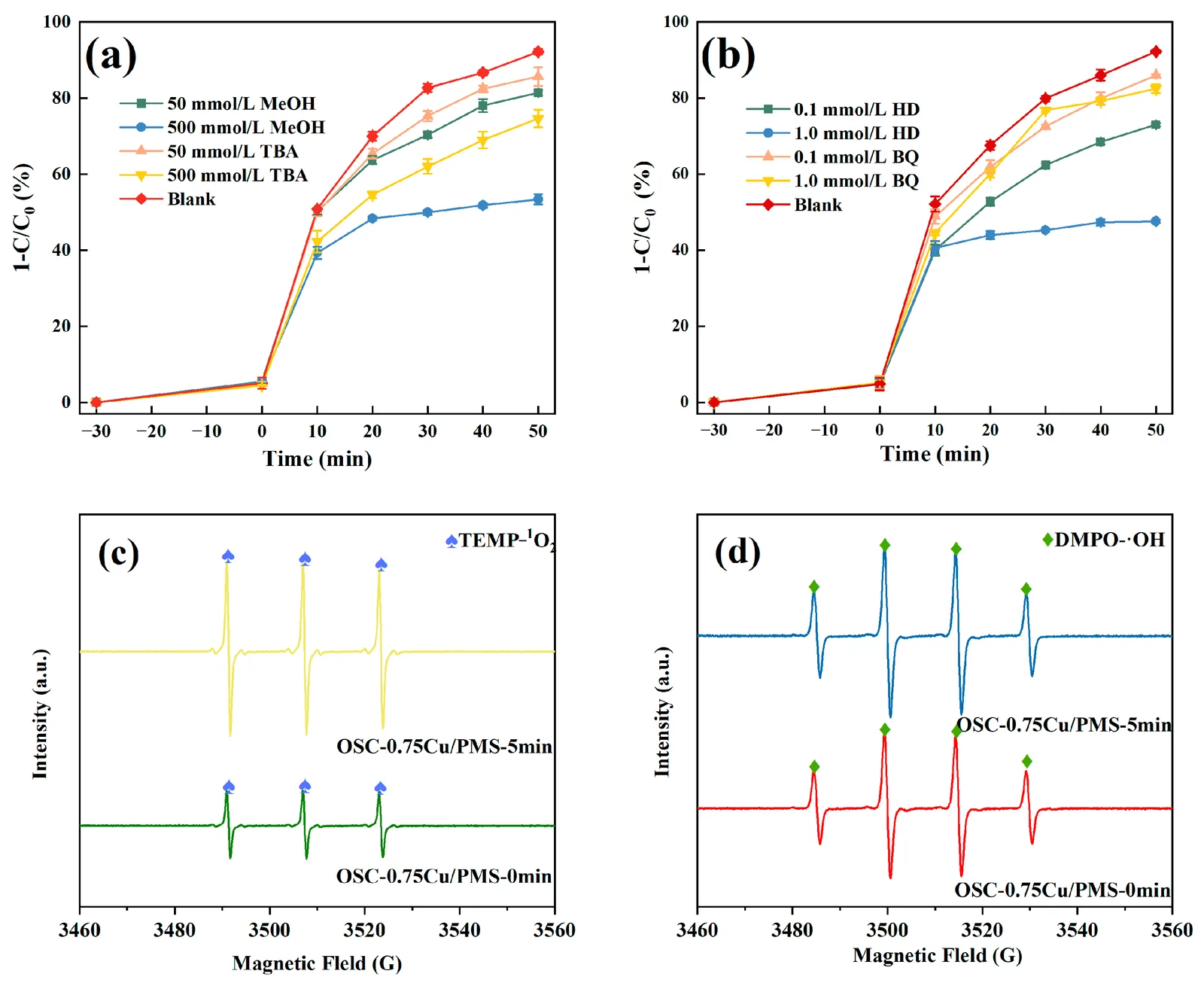
Effect of different inhibitors on degradation of PNP: (**a**) MeOH and TBA, (**b**) HD and BQ. Spin-trapping EPR signals of (**c**) TEMP-^1^O_2_ and (**d**) DMPO-•OH.

Figure 9a shows the inhibitory effects of TBA and MeOH on PNP degradation. TBA exhibited weak inhibition; 50 mmol/L TBA reduced the degradation efficiency by 6.6%, while 500 mmol/L TBA reduced it by 17.57%. In contrast, MeOH showed stronger inhibition; 50 mmol/L MeOH reduced the degradation efficiency by 10.86%, while 500 mmol/L MeOH reduced it by 38.88%. These results indicate that •SO_4_^−^ and •OH made a partial contribution to the PNP degradation process.

Figure 9b shows the inhibitory effects of HD and BQ on PNP degradation. BQ exhibited weak inhibition; 0.1 mmol/L BQ reduced the degradation efficiency by 6.3%, while 1.0 mmol/L BQ reduced it by 9.79%. Conversely, HD exhibited strong inhibition; 0.1 mmol/L HD reduced the degradation efficiency by 19.24%, while 1.0 mmol/L HD reduced it by 44.66%. These results suggest that ^1^O_2_ played a dominant role in the degradation of PNP by the OSC-0.75Cu/PMS system. Quenching experiments revealed that four ROS, •OH, •SO_4_^−^, •O_2_^−^, and ^1^O_2_, were generated in the system, with •OH, •SO_4_^−^, and ^1^O_2_ being the primary contributors.

To further verify the presence of reactive species, EPR spectroscopy was employed to detect the ROS generated at 0 and 5 min after the reaction initiation. As shown in Figure 9c, a typical 1:1:1 triplet signal of the TEMP-^1^O_2_ adduct was observed in the EPR spectrum, indicating the generation of ^1^O_2_ in the system [56,57]. Combined with the O 1s XPS results, defect-related oxygen sites may contribute to PMS adsorption and activation and facilitate ^1^O_2_ generation [58]. Additionally, ^1^O_2_ can also form through self-decomposition of •SO_5_^−^ (Equations (8) and (9)) [50].

Cu(II) + HSO_5_^−^ → Cu(I)+•SO_5_^−^ + H^+^, (8)

2•SO_5_^−^ → S_2_O_8_^2−^ + ^1^O_2_. (9)

Additionally, characteristic peaks corresponding to the DMPO-•OH adduct were observed in Figure 9d, confirming the presence of •OH in the system. No distinct peaks corresponding to DMPO-•SO_4_^−^ were detected, likely due to the rapid reaction of •SO_4_^−^ with water to generate •OH (Equation (10)), and the DMPO-•SO_4_^−^ signal can also be converted to DMPO-•OH via a nucleophilic substitution reaction [58,59].

SO_4_^−^ + H_2_O → SO_4_^2−^ + •OH + H^+^. (10)

### 3.3. LC–MS Analysis of PNP Transformation Products

**Figure 10:**
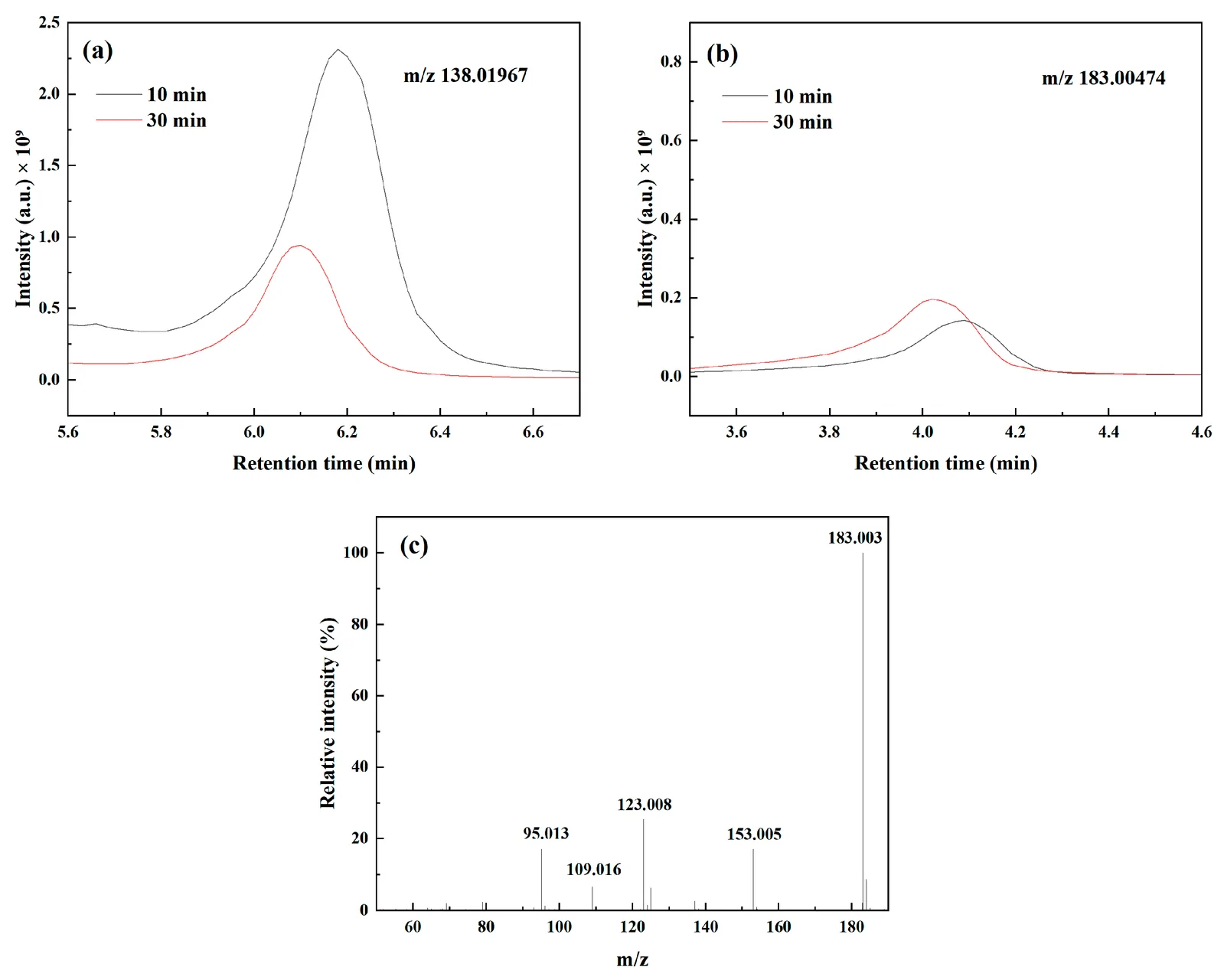
LC–MS analysis of PNP transformation in the OSC-0.75Cu/PMS system: EICs of (**a**) *m*/*z* 138.01967 and (**b**) *m*/*z* 183.00474 at 10 and 30 min. (**c**) MS/MS spectrum of the precursor ion at *m*/*z* 183.004.

To investigate the transformation products generated during PNP degradation, reaction samples collected at 10 and 30 min were analyzed by LC–MS in the negative-ion mode. As shown in Figure 10a, the EIC at *m*/*z* 138.01967, assigned to deprotonated PNP, decreased substantially from 10 to 30 min. In contrast, the signal at *m*/*z* 183.00474, detected at a retention time of approximately 4.07–4.09 min, increased with reaction time (Figure 10b), indicating the accumulation of a transformation product.

The accurate mass of the ion at *m*/*z* 183.00474 was consistent with the [M−H]^−^ ion of C_6_H_4_N_2_O_5_. Its MS spectrum showed major fragment ions at *m*/*z* 153.005, 123.008, 109.016, and 95.013 (Figure 10c). Accordingly, this ion was tentatively assigned to a nitrophenol-type transformation product. However, because no authentic standard was available, the exact positional isomer could not be conclusively identified. These results provide evidence for the formation of transformation products during PNP degradation but do not demonstrate complete mineralization.

### 3.4. Degradation Mechanism

**Figure 11:**
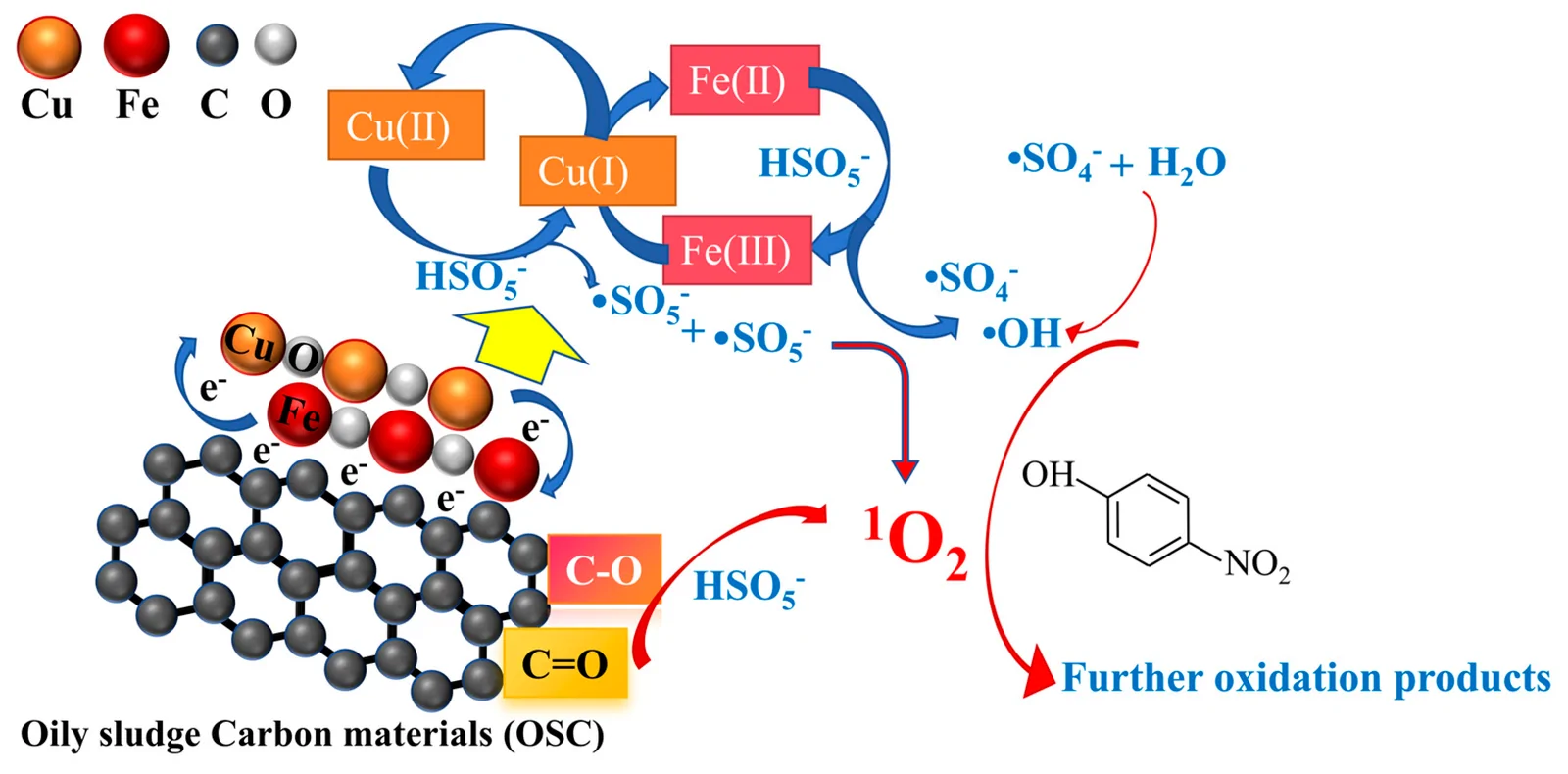
Proposed mechanism of PNP removal in the OSC-0.75Cu/PMS system.

The XPS results suggest a possible interfacial redox interaction between Cu and Fe species during PMS activation. However, the detailed electron-transfer pathway remains to be further verified. Based on the above results and analysis, we proposed possible reaction mechanisms. During the reaction, surface Cu(I) reduces Fe(III) to Fe(II) and is oxidized to Cu(II). Meanwhile, Fe(II) activates PMS to generate •SO_4_^−^ and •OH [60] (Equations (11) and (12)), whereas Cu(II) and Cu(I) activate PMS to produce •SO_5_^−^ and •SO_4_^−^, respectively (Equations (8) and (13)) [61]. Subsequently, •SO_4_^−^ reacts with water to form •OH (Equation (10)). Defect-related oxygen sites may also contribute to PMS adsorption and activation and facilitate ^1^O_2_ generation. Additionally, self-decomposition of •SO_5_^−^ constitutes another pathway for ^1^O_2_ generation [26] (Equations (8) and (9)). C=O and C—O on the activated carbon support surface also act as active sites, activating PMS to generate ^1^O_2_ (Equations (6) and (7)) (Figure 11).

Fe(II) + HSO_5_^−^ → Fe(III) + •SO_4_^−^ + OH^−^, (11)

Fe(II) + HSO_5_^−^ → Fe(III) + •OH + SO_4_^2−^, (12)

Cu(I) + HSO_5_^−^ → Cu(II) + •SO_4_^−^ + OH^−^, (13)

## 4. Materials and Methods

### 4.1. Materials

p-Nitrophenol (PNP, CAS 100-02-7), potassium peroxymonosulfate (CAS 10058-23-8), copper(II) nitrate trihydrate (CAS 10031-43-3), p-benzoquinone (BQ, CAS 106-51-4), L-histidine (HD, CAS 71-00-1), methanol (MeOH, CAS 67-56-1), and tert-butanol (TBA, CAS 75-65-0) were purchased from Shanghai Macklin Biochemical Technology Co., Ltd. (Shanghai, China). Anhydrous ethanol (CAS 64-17-5), sodium chloride (CAS 7647-14-5), sodium sulfate (CAS 7757-82-6), sodium bicarbonate (CAS 144-55-8), acetic acid (CAS 64-19-7), sodium nitrate (CAS 7631-99-4), sodium hydroxide (CAS 1310-73-2), and sulfuric acid (CAS 7664-93-9) were obtained from Sinopharm Chemical Reagent Co., Ltd. (Shanghai, China). Fulvic acid (CAS 479-66-3) was purchased from Shanghai Aladdin Biochemical Technology Co., Ltd. (Shanghai, China). All chemicals were of analytical grade and were used without further purification.

### 4.2. Characterization

The crystal structure was analyzed using a Bruker D8 Advance X-ray diffractometer (Billerica, MA, USA) with a Cu Kα radiation source (λ = 0.15406 nm), operating at a tube voltage of 40 kV and a tube current of 40 mA. The obtained data were analyzed using Jade 6.0 software. The surface morphology and elemental distribution were investigated using a Zeiss Gemini SEM 300 field emission scanning electron microscope (Oberkochen, Germany) with energy-dispersive X-ray spectroscopy (EDS). FTIR spectra were collected using a Thermo Scientific Nicolet iS20 spectrometer (Waltham, MA, USA) in the wavenumber range of 4000–400 cm^−1^ at a resolution of 4 cm^−1^ with 32 scans. X-ray photoelectron spectroscopy (XPS) was performed on a Thermo Scientific K-Alpha spectrometer (Waltham, MA, USA) to analyze the chemical states of the samples before and after the reaction, operating at a working voltage of 12 kV and a filament current of 6 mA. Reactive species generated in the catalytic system were identified using a Bruker EMXplus-6/1 electron paramagnetic resonance (EPR) spectrometer (Bruker BioSpin, Ettlingen, Germany), with 2,2,6,6-tetramethylpiperidine (TEMP) and 5,5-dimethyl-1-pyrroline N-oxide (DMPO) employed as spin-trapping agents.

### 4.3. Synthesis of Catalyst

#### 4.3.1. Self-Loaded Iron Oxide OSC

The OSC was prepared using oil sludge (OS) from our previous study [22]. Briefly, OS was pyrolyzed at 800 °C (1 h, N_2_), cooled, and ground to obtain OSC material. According to our previous findings, the raw oil sludge contains intrinsic iron species, leading to the formation of iron oxide species within the carbon matrix after pyrolysis. Therefore, no external iron sources were introduced in this study. Detailed profiling of other residual organic contaminants and trace metals in the raw oil sludge was beyond the scope of this study and should be considered in future scale-up assessments.

#### 4.3.2. Copper Oxide-Loaded OSC

A mixture of 1 g of OSC and *X* g of Cu(NO_3_)_2_·3H_2_O was prepared by grinding in a ceramic mortar for 20 min. The mixture was then calcined at 500 °C for 1 h under N_2_ atmosphere. After cooling, the catalyst was sequentially washed with anhydrous ethanol and deionized water and then vacuum-dried at 60 °C for 12 h. After cooling, the product was ground into a powder to obtain the OSC-*X*Cu catalyst. The mass ratios of Cu(NO_3_)_2_·3H_2_O to OSC were 1:1, 1:0.75, and 1:0.5, and the resulting samples were labeled OSC-1.0Cu, OSC-0.75Cu, and OSC-0.5Cu, respectively.

### 4.4. Experimental Procedure

The degradation experiments of p-nitrophenol (PNP) were carried out in a multi-channel photoreactor (Beijing Perfectlight Technology Co., Ltd., Beijing, China). The procedure was as follows: The catalyst was added to 50 mL PNP solution in a quartz glass reactor and stirred for 30 min to achieve adsorption–desorption equilibrium. Subsequently, PMS was then added to initiate the degradation experiment. At each sampling time intervals, 1.0 mL of the reaction solution was withdrawn, and 0.1 mL of methanol was added to quench the reaction. Except for the pH effect experiments, in which H_2_SO_4_ and NaOH solutions were used to adjust the pH to the desired value, no pH adjustment was performed in other experiments. The baseline experimental conditions were as follows: catalyst dosage = 1.0 g/L, initial PNP concentration = 20 mg/L, PMS concentration = 1.0 mmol/L, initial temperature = 25 ± 2 °C, and stirring rate = 430 rpm. An initial PNP concentration of 20 mg/L was selected as a representative intermediate concentration within the tested range of 10–40 mg/L, providing an appropriate basis for comparing the effects of the other operating parameters. Variations in these conditions for investigating the effects of individual factors are described in the corresponding sections.

The PNP concentration was determined using an LC-16 high-performance liquid chromatography (HPLC) system (Shimadzu (Suzhou) Instruments Manufacturing, Co., Ltd., Suzhou, China). The HPLC conditions were as follows: a C18 reversed-phase column (4.6 mm × 250 mm, 5 μm, Shimadzu, Nakagyo-ku, Japan) with a column temperature of 35 °C. The mobile phase consisted of methanol and 5.1% (*v*/*v*) acetic acid aqueous solution (63:37, *v*/*v*) at a flow rate of 1.0 mL/min, and the detection wavelength was set at 312 nm. After separation of the catalyst, the dissolved Cu and Fe concentrations in the supernatant were determined spectrophotometrically using the neocuproine and 1,10-phenanthroline methods at 457 and 510 nm, respectively. To identify possible transformation products, reaction samples collected at 10 and 30 min were filtered through a 0.22 μm membrane filter and analyzed using an Ultimate 3000 UHPLC system coupled with a Q-Exactive mass spectrometer (Thermo Scientific, Waltham, MA, USA) in negative electrospray ionization mode with a C18 column. Full-scan spectra were acquired over an *m*/*z* range of 50–600 at a resolution of 70,000, while MS/MS spectra were obtained at a resolution of 17,500 using normalized collision energies of 30, 60, and 90.

## 5. Conclusions

In this work, a copper oxide and iron oxide co-loaded oil sludge-derived carbon catalyst was successfully synthesized. The OSC-0.75Cu/PMS system achieved 96.7% removal of 10 mg/L PNP within 50 min. C=O and C—O groups on the carbon material surface can activate PMS to generate ^1^O_2_. In addition, the coexistence of Cu and Fe species may facilitate surface redox reactions during PMS activation, while defect-related oxygen sites may contribute to ^1^O_2_ generation. Multiple reactive species, including •OH, •SO_4_^−^, •O_2_^−^, and ^1^O_2_, all contributed to the oxidative degradation, with ^1^O_2_ playing the dominant role and •O_2_^−^ contributing the least.

The OSC-0.75Cu/PMS system retained appreciable PNP degradation in the tested real water matrices. Nevertheless, the decline in activity upon reuse, along with the non-negligible leaching of Cu and Fe, suggests that catalyst immobilization, regeneration, and long-term continuous-flow evaluation are necessary before practical application.

## Data Availability

Data are contained within the article.
